# Anti-Inflammation and Anti-Oxidation: The Key to Unlocking the Cardiovascular Potential of SGLT2 Inhibitors and GLP1 Receptor Agonists

**DOI:** 10.3390/antiox13010016

**Published:** 2023-12-20

**Authors:** Veronika A. Myasoedova, Michele Bozzi, Vincenza Valerio, Donato Moschetta, Ilaria Massaiu, Valentina Rusconi, Daniele Di Napoli, Michele Ciccarelli, Valentina Parisi, Piergiuseppe Agostoni, Stefano Genovese, Paolo Poggio

**Affiliations:** 1 Centro Cardiologico Monzino IRCCS, 20138 Milan, Italy; veronika.myasoedova@ccfm.it (V.A.M.); michele.bozzi@ccfm.it (M.B.); vincenza.valerio@ccfm.it (V.V.); donato.moschetta@ccfm.it (D.M.); ilaria.massaiu@ccfm.it (I.M.); valentina.rusconi@ccfm.it (V.R.); piergiuseppe.agostoni@ccfm.it (P.A.); stefano.genovese@ccfm.it (S.G.); 2 Department of Medicine, Surgery and Dentistry, University of Salerno, 84084 Fisciano, Italy; ddinapoli@unisa.it (D.D.N.); mciccarelli@unisa.it (M.C.); 3 Department of Translational Medical Sciences, Federico II University, 80138 Naples, Italy; valentina.parisi@unina.it

**Keywords:** sodium-glucose cotransporter 2 inhibitors, glucagon-like peptide 1 receptor agonists, cardio-metabolism, oxidative stress, inflammation, fibrosis, epicardial adipose tissue

## Abstract

Type 2 diabetes mellitus (T2DM) is a prevalent and complex metabolic disorder associated with various complications, including cardiovascular diseases. Sodium-glucose co-transporter 2 inhibitors (SGLT2i) and glucagon-like peptide 1 receptor agonists (GLP1-RA) have emerged as novel therapeutic agents for T2DM, primarily aiming to reduce blood glucose levels. However, recent investigations have unveiled their multifaceted effects, extending beyond their glucose-lowering effect. SGLT2i operate by inhibiting the SGLT2 receptor in the kidneys, facilitating the excretion of glucose through urine, leading to reduced blood glucose levels, while GLP1-RA mimic the action of the GLP1 hormone, stimulating glucose-dependent insulin secretion from pancreatic islets. Both SGLT2i and GLP1-RA have shown remarkable benefits in reducing major cardiovascular events in patients with and without T2DM. This comprehensive review explores the expanding horizons of SGLT2i and GLP1-RA in improving cardiovascular health. It delves into the latest research, highlighting the effects of these drugs on heart physiology and metabolism. By elucidating their diverse mechanisms of action and emerging evidence, this review aims to recapitulate the potential of SGLT2i and GLP1-RA as therapeutic options for cardiovascular health beyond their traditional role in managing T2DM.

## 1. Introduction

In the clinical scenario, two new classes of drugs have emerged as potential game-changers in the treatment of type 2 diabetes mellitus (T2DM). These drugs are known as sodium-glucose co-transporter 2 inhibitors (SGLT2i) and glucagon-like peptide 1 receptor agonists (GLP1-RA). While their primary purpose is to lower blood glucose levels in patients with T2DM, recent studies have shown that they offer several other benefits beyond their glucose-lowering effect.

To understand how these drugs work, let us take a closer look at their mechanisms of action. SGLT2i operate within the kidneys, inhibiting the SGLT2 receptor responsible for glucose reabsorption. By doing so, SGLT2i facilitate the excretion of glucose in the urine, effectively lowering blood glucose levels. Meanwhile, GLP1-RA mimic the action of the hormone GLP1, which stimulates glucose-dependent insulin secretion from the pancreatic islets. This means that when glucose levels rise after a meal, the body is induced to produce extra insulin to control the increase.

As scientists and medical professionals continue to study SGLT2i and GLP1-RA, they are discovering that these drugs offer more than just antidiabetic effects. Indeed, an increasing number of studies and trials are showing that both categories of drugs exhibit a wide range of application prospects that go beyond diabetes treatment.

One of the most significant side benefits of SGLT2i is its ability to improve metabolic control. Studies have shown that these drugs induce a fasting response, leading to body weight loss [1]. Additionally, SGLT2i has been found to modulate tissue inflammation and contribute to renal protection, preserving kidney function in the long term and promoting osmotic diuresis and natriuresis [1]. These drugs prevent both hyper and hypoglycemia and protect kidney function from failing via blood glucose-dependent and -independent mechanisms [1].

GLP1-RA, on the other hand, has also been found to offer a wealth of benefits beyond its antidiabetic effects. These metabolic regulators have been shown to facilitate body weight loss and offer pancreatic protection by promoting islet β-cell proliferation. Furthermore, evidence has proven their neuroprotective role in reducing neuroinflammation, protecting neurons against metabolic and oxidative insults, and promoting nerve growth [2].

However, among all the benefits associated with SGLT2i and GLP1-RA, probably the most interesting evidence that unites the two categories of drugs is their significant reduction of major cardiovascular events in patients with T2DM. Studies have shown that these drugs offer cardioprotection in patients with established atherosclerotic cardiovascular disease, reduce the risk of admission to the hospital for heart failure, and lower cardiovascular and all-cause mortality [3]. Subsequent studies further investigated and demonstrated that the cardioprotective effects of these drugs were not restricted to patients with T2DM but were also observed in patients without the condition [3].

In this review, we will explore the potential of SGLT2i and GLP1-RA in improving cardiovascular health, focusing on the latest research on these drugs and their effects on the heart, physiology, and metabolism (Table 1).

## 2. Cardio-Metabolism

**Figure 1:**
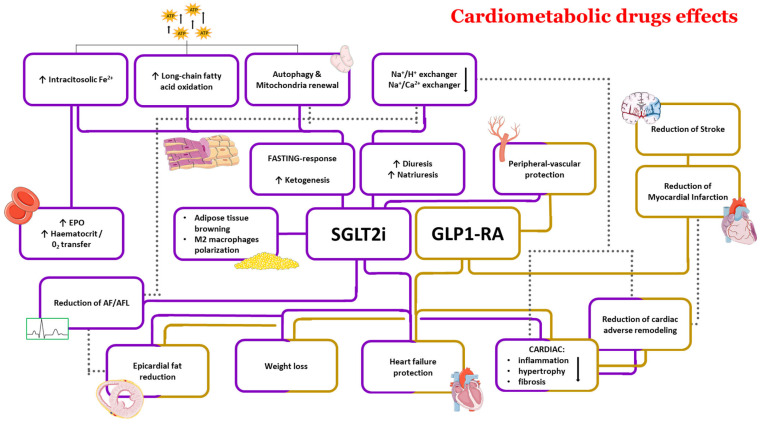
SGLT2i and GLP1-RA cardiometabolic effects. The violet boxes represent the action of SGLT2i, while the yellow boxes represent the action of GLP1-RA. The arrow pointing downwards reflect a downregulation of the mechanisms, while upwards arrows indicate an upregulation of the mechanisms. AF: atrial fibrillation; AFL: atrial flutter; EPO: erythropoietin; GLP1-RA: glucagon-like peptide 1 receptor agonist; SGLT2i: sodium-glucose co-transporter 2 inhibitors.

In this chapter, we will explore the additional effects of SGLT2i and GLP1-RA beyond their glucose-lowering role in patients with T2DM. These two classes of drugs have demonstrated impressive cardiometabolic outcomes, including improvements in energy homeostasis, weight loss, and blood pressure reduction. We will provide a comprehensive summary of these effects and their potential implications for the management of T2DM, as well as their possible benefits in the treatment of other metabolic disorders (Figure 1).

### 2.1. Oxidative Stress, Inflammation, Cardiac Fibrosis, and Remodeling

Oxidative stress and chronic inflammation induce impaired metabolic homeostasis, leading to decreased peripheral insulin sensitivity, β-cell dysfunction, and the development of diabetic cardiomyopathy [4]. Meanwhile, hyperglycemia and lipotoxicity are associated with the increased production of reactive oxygen species (ROS) or reactive nitrogen species (RNS) [4]. Excessive ROS production can deteriorate the formation of advanced glycation end products (AGEs), activate receptors for AGEs (RAGE) and protein kinase C (PKC) isoforms, increase the flux of the hexosamine pathway, and activate the NF-κB pathway [5], indirectly leading to diabetic cardiomyopathy [6].

In vitro experiments show that SGLT2i alone or combined with GLP1-RA decreased cell death and oxidative stress via improved NOS activity and increased NO production in cardiac muscle cells [7]. SGLT2i had cytoprotective properties in endothelial cells; indeed, both empagliflozin and dapagliflozin restored NO bioavailability by inhibiting ROS generation in TNFα-stimulated cells [8,9]. In addition, SGLT2i can prevent cardiac dysfunction by improving oxidative stress and mitochondrial dysfunction in animal models [10] via the stimulation of Nrf2/ARE signaling and suppression of the TGF-β/Smad pathway [11], preventing hydrogen peroxide release and preserving ATP production [12]. Other studies indicated that SGLT2i can moderate oxidative stress and reduce the expression of NADPH oxidase subunits such as NOX2, p22phox, and p47phoxas well as the urinary excretion of 8-hydroxy-2′-deoxyguanosine (8-OHdG), a marker of oxidative DNA damage [13].

Recent clinical studies have demonstrated the ability of SGLT2i to reduce oxidative stress by measuring plasma levels of 8-iso-PGF2α that decreased 24 weeks after SGLT2i treatment in newly diagnosed T2DM patients [14]. Meanwhile, a pilot study measuring the blood levels of soluble NADPH oxidase 2 (NOX2)-derived peptide, which is a marker of NOX2 activation and hydrogen peroxide production in patients with T2DM, showed that SGLT2i significantly reduced the level of both markers [15].

Regarding the effect of GLP1-RA on oxidative stress, it is known that a novel oral GLP-1RA hypoglycemic peptide 2 (OHP2) and exendin-4 inhibited neutral lipid accumulation as well as intracellular and mitochondrial ROS generation in palmitate- or methylglyoxal-induced rat cardiomyocytes [16,17,18]. In addition, GLP1-RA ameliorated IL-1β-induced ROS production and NOX-4 expression in cardiomyocytes [19]. In animal models, GLP1-RA can improve myocardial oxidative stress through suppression of NOX-4, with a concomitant increase in superoxide dismutase 1 (SOD-1) and glutathione peroxidase [20]. Additionally, GLP1-RA can reduce myocardial triglyceride and diacylglycerol levels, NOX activity, and oxidative stress through activation of the AMPK-Sirt1 pathway [21]. A clinical report also indicates that plasma levels of 8-iso-PGF2α are reduced after 26 weeks of GLP1-RA administration in patients with T2DM, suggesting a reduction in oxidative stress through reduced ROS production and increased antioxidant capacity, partly independent of the glucose-lowering effect [22].

Cardiac inflammation and fibrosis are the primary pathological mechanisms operating in the failing heart, irrespective of the underlying etiology of heart failure [23]. SGLT2i and GLP1-RA are emerging as promising therapeutic options for mitigating these mechanisms. Numerous studies have demonstrated significant anti-inflammatory and anti-fibrotic effects of both classes of drugs, both in vitro and in vivo [24,25].

In vitro studies on human fibroblasts have shown that the SGLT2i induce a quiescent-like phenotype, reduce the extracellular matrix remodeling response, and decrease the expression of profibrotic markers [26]. In mouse models of heart failure, administration of SGLT2i has been found to induce anti-inflammatory effects on the heart without any changes in the levels of circulating ketone bodies or cardiac ATP production, suggesting a more direct involvement. Specifically, a lower cardiac and peripheral activation of NLRP3 inflammasome and ROS production was observed [27]. In parallel, other studies on similar murine models have demonstrated cardiac benefits, such as prevention of ejection fraction reduction, improved diastolic function, and reduced wall stress, without significant changes in fibrosis processes, suggesting that anti-fibrotic effects are not necessarily correlated [28,29].

Another in vivo study on heart failure evaluated the effects of both SGLT2i and GLP1-RA, with the latter showing more significant effects in attenuating cardiometabolic dysregulation and improving cardiac structure and function [25]. Specifically, GLP1-RA demonstrated significant effects in reducing cardiac hypertrophy and myocardial fibrosis, as well as lowering atrial weight, natriuretic peptide levels, and lung congestion. SGLT2i also improved inflammation and tissue fibrosis but with less pronounced effects on functional abnormalities and structural remodeling [25].

Inflammation and oxidative stress caused by diabetes can also lead to myocardial apoptosis and diabetic cardiomyopathy, which is characterized by progressive cardiac remodeling [30]. In this context, increased fibrosis occurs in association with myocyte loss, further exacerbating myocardial stiffness and eventually leading to cardiac diastolic and systolic dysfunction [30]. In diabetic animal models, GLP1-RA inhibited oxidative stress and significantly reduced mitochondrial morphological alterations, with a significant reduction in collagen deposition [30]. In addition, in rats with MI-induced chronic heart failure, GLP1 treatment showed the capability to arrest adverse cardiac remodeling [31].

These pathological changes contribute to the development and progression of heart failure in diabetic individuals and highlight the need for effective therapeutic interventions to address the underlying mechanisms. In this regard, GLP1-RA have demonstrated significant potential for preventing adverse cardiac remodeling in diabetic individuals. Indeed, the pre-treatment with GLP1 (7–36) amide can protect the heart against ischemic left ventricular dysfunction and improve its recovery during reperfusion [32]. Moreover, chronic therapy with GLP-1 RA and/or SGLT-2i has a positive effect on the clinical outcomes of patients with diabetes mellitus who are hospitalized due to acute myocardial infarction [33].

SGLT2i have also been studied for their involvement in reverse cardiac remodeling. One study, in particular, demonstrated that patients with T2DM and established cardiovascular disease who were treated with SGLT2i for three months showed a significant reduction in left ventricular mass, which was associated with a beneficial outcome on diastolic function [34].

However, further investigations are needed to fully understand the effectiveness of SGLT2i and GLP1-RA in promoting reverse cardiac remodeling and attenuating the inflammatory and fibrotic processes underlying heart failure, particularly in the context of diabetes.

### 2.2. Epicardial Fat

Epicardial adipose tissue (EAT) is a visceral fat depot located between the heart and the pericardium. It shares many pathophysiological properties with other visceral fat depots, including local inflammation [35]. Increasing evidence suggests that EAT plays an important role in cardiometabolic risk and is involved in the progression of coronary artery disease (CAD), heart failure, and atrial fibrillation (AF) [36,37]. Since EAT may be a modifiable risk factor, it represents a potential therapeutic target for drugs that provide cardiovascular benefits. In diabetes, EAT is significantly thicker compared to controls and has a greater degree of inflammation, which is associated with systolic-diastolic dysfunction, CAD, worse cardiopulmonary fitness, and higher mortality [38].

SGLT2i and GLP1-RA have been shown to promote weight loss and alter fat tissue homeostasis, suggesting that they might potentially target epicardial fat dysregulation and related cardiovascular risk factors. Both SGLT2i and GLP1-RA have been tested in targeted clinical studies and have shown a significant beneficial effect on EAT reduction within three months, with even better stabilization beyond six months of administration [39]. These drugs could represent a therapeutic strategy for dysfunctional EAT, particularly for young patients with a high body mass index (BMI) [39].

Consistent with previous findings, the GLP1-RA also seem to exert a browning effect on EAT [40], similar to what occurs in visceral fat. This process further contributes to the recovery of EAT fitness, as brown fat activation increases energy consumption and fat depot depletion, potentially resulting in the restoration of physiological epicardial volume.

All the effects of SGLT2i and GLP1-RA on EAT could represent an additional avenue for the treatment of cardiovascular impairments directly or indirectly involved with epicardial fat dysfunction, whether in the presence or absence of diabetes. More pooled data will be needed to strengthen this possibility further.

### 2.3. Arrhythmia and Fibrillation

The compelling evidence from clinical trials consistently demonstrates the effectiveness of these agents in mitigating hospitalizations for heart failure as well as cardiovascular morbidity and mortality [41]. Simultaneously, numerous investigations have explored the potential therapeutic benefits of SGLT2i on cardiac electrophysiological abnormalities.

Recent findings have shed light on the noteworthy effects of SGLT2i in opposing arrhythmias and AF. The first meta-analysis of large-scale cardiovascular outcome trials demonstrated that SGLT2i significantly decreased the incidence rates of AF in patients with T2DM, heart failure, or chronic kidney disease while having no significant impact on stroke risk for both diabetic and non-diabetic patients [42]. Another study investigated the effect of SGLT2i on AF and atrial flutter (AFL), which are considered intermediate markers of stroke risk. The results consistently indicated that SGLT2i could prevent both AF and AFL without significantly affecting the overall stroke risk [43]. Further investigations from large clinical trials have confirmed a significant reduction in AF incidence, with benefits for ventricular arrhythmias as well. Ongoing clinical trials are expected to provide further supporting evidence in the coming years. Along with clinical trials, animal models (primarily rodents) and cellular models have also yielded confirmatory evidence [44].

Further investigation is required to fully comprehend the molecular mechanisms underlying the anti-arrhythmic effect of SGLT2i. Nonetheless, experimental and clinical data suggest possible explanations for the cardioprotective benefits of these drugs, including both direct and indirect effects on the heart. Firstly, SGLT2i increase diuresis, glucose, and sodium excretion, resulting in a reduction of plasma volume, atrial blood pressure, and atrial dilation effects [45]. This attenuated overload may protect the heart from dysfunctional activity. Furthermore, in diabetic patients, the upregulation of the sodium-hydrogen exchanger leads to an increase in intracellular sodium content, higher Na^+^/Ca^2+^ exchanger activity, and higher calcium levels in the sarcoplasmic reticulum [46]. The augmented natriuresis induced by SGLT2i, accompanied by the suppression of the sodium-hydrogen exchange, reduces cardiac intracellular sodium and calcium levels. This correlates with a reduction in myocardial hypertrophy, fibrosis, and adverse remodeling, thereby reducing the risk of arrhythmia [42]. In addition, this mechanism helps to stabilize mitochondrial activation of the Na^+^/Ca^2+^ exchanger, preventing mitochondrial damage, regardless of the presence of diabetes [42,44].

Another proposed effect of SGLT2i is related to the increased volume of EAT in patients with T2DM, which leads to localized inflammation via adipokines and possibly increases the incidence of AF [47]. The role of SGLT2i in reducing EAT, as discussed previously, may contribute to their anti-arrhythmic action [42,44]. However, several other mechanisms have been proposed, ranging from SGLT2i playing a role in sympathetic inhibition and autonomic cardiovascular regulation [1,42] to a hypothetical direct interaction of these compounds with atrial myocardium, which may ameliorate atrial electrical and structural remodeling [44].

Recent studies have evaluated the correlation between glucose-lowering agents and the risk of AF/AFL, comparing SGLT2i and GLP1-RA [48]. As mentioned earlier, AF and AFL are associated with increased morbidity (i.e., heart failure and stroke) and mortality in patients with T2DM [43,48]. SGLT2i showed the strongest protection against incident AF/AFL while not improving stroke outcomes. On the other hand, GLP1-RA have demonstrated a reduction in stroke but does not significantly prevent the onset of AF/AFL [49]. However, GLP1-RA increased the heart rate and the number of adverse cardiac events [50].

Overall, further research is needed to explore all these findings, as the role of these new classes of cardiometabolic drugs is still poorly investigated at the onset of arrhythmia.

### 2.4. Haematocrit, Erythropoietin, and Iron Homeostasis

SGLT2 inhibition has been observed to have an interesting effect on hematocrit levels. In the context of heart failure, low hematocrit is a common occurrence, which can arise from either haemodilution characterized by an increase in plasma volume or anemia that is marked by a decrease in red blood cell volume [51]. Patients with anemia have been found to have a poor prognosis for heart failure, while those with hemodilution experience even worse outcomes [51]. These findings suggest that volume overload may significantly contribute to the adverse outcomes observed in these individuals [51].

The potential benefits of SGLT2i on hematocrit levels could offer an alternative strategy for managing heart failure patients. SGLT2i promote mild osmotic diuresis and natriuresis, leading to a reduction in blood pressure and relative volume contraction. Consequently, this indirectly increases hematocrit levels and reduces the volume overload imposed on the heart [1]. In addition, there is evidence to suggest a direct effect of SGLT2i on hematocrit levels [52,53]. Studies have shown that SGLT2i increase erythropoietin (EPO) production, erythropoiesis, and hematocrit in individuals with T2DM [54]. Although it is assumed that the increased EPO production is related to the improved renal function resulting from this drug class, there may be a more direct mechanism that has yet to be identified [1,54,55]. Nonetheless, EPO has been shown to improve oxygen delivery and promote the regeneration of pro-vascular progenitor cells in the bone marrow, leading to enhanced myocardial efficiency [1]. SGLT2i are believed to further improve this efficiency through their potential effects on iron homeostasis [54]. Specifically, they have been suggested to decrease the levels of ferritin and hepcidin, which are typically elevated in heart failure, thereby increasing the availability of bio-reactive cytosolic Fe^2+^ [54]. This form of iron is used for the synthesis of hemoglobin in erythrocytes and ATP in cardiomyocytes, providing further energy improvement and attenuating the state of inflammation mediated by iron deficiency in the failing heart [56]. These findings underlie the broad potential benefits of cardiometabolic drugs in protecting against heart failure and improving cardiovascular outcomes.

### 2.5. Metabolic Changes

SGLT2i were designed to promote glucose excretion through urine, resulting in increased glycosuria and subsequent reductions in glucose and insulin levels. This leads to a state of lowered intracellular glucose availability, similar to a starvation-like state [57]. In response to carbohydrate deprivation, ketogenesis is triggered, which physiologically compensates for the decreased sugar reserves. The ketogenic response enhances the mobilization of free fatty acids (FFA) from adipose tissue stores and promotes the tissue-specific enhancement of fatty β-oxidation in the liver [57]. The increased utilization of FFA leads to an increment in lipolysis, promoting a reduction in inflammation mediated by adipose tissue, decreased oxidative stress and its damages, and reduced levels of circulating uric acid [57,58].

Numerous in vivo studies have confirmed the metabolic changes induced by SGLT2i and revealed several side benefits [59]. For instance, studies on diet-induced obese mouse models indicated that the metabolic shift towards ketone bodies, induced by SGLT2i, was associated with the polarization of M1 to M2 macrophages in adipose tissue, along with a significant fat-browning effect [59]. M2-polarized macrophages generated anti-inflammatory cytokines, reducing both obesity-induced inflammation and insulin resistance in fat and liver tissues, thereby promoting the recovery of normal energy homeostasis [59,60,61]. Furthermore, several studies identified a browning process in white adipose reserves, resulting in a metabolically active tissue responsible for thermogenesis and the consumption of surplus calories [59,60]. Other studies on murine hearts suggested that SGLT2i-induced ketogenesis may provide extra fuel, increasing cardiac efficiency [62]. However, whether this effect operates in humans as well needs to be further investigated.

Recent studies have suggested a potential interaction between SGLT2i and glucose transporter 1 (GLUT1) [63], which normally facilitates the transport of glucose across plasma membranes in almost all tissues. By inhibiting GLUT1, SGLT2i obstruct glucose transport within cells and contributes to intracellular nutrient-deprivation signaling. The metabolic responses to this obstruction are cellular autophagy and mitochondrial biogenesis stimulation, both in the diabetic kidney and heart [1,56]. Autophagy is a cellular process that clears cells from dysfunctional organelles as well as toxic glucose and lipid by-products [56]. In cardiomyocytes, SGLT2i promote autophagy as a systemic cellular defense and pro-survival mechanism to the starvation condition. The consequence is the disposal of dysfunctional mitochondria, replaced by healthy ones, leading to reduced oxidative stress and higher ATP synthesis. Moreover, the autophagic clearance of glucose and lipid wastes prevents endoplasmic reticulum stress, cardiomyocyte apoptosis, and pro-inflammatory signals [56].

ATP synthesis is crucial for the proper functioning of cardiomyocytes, and its production is impaired in failing hearts [64]. Therefore, ATP recovery is critical in slowing down the progression of heart failure, making it a game-changer. As anticipated, SGLT2i induce a recovery shift of metabolism from glucose to FFA, enhancing their transport and β-oxidation in the heart [57]. This process is biochemically profitable, as it provides a quantity of ATP three times higher than glucose oxidation and 20 times higher than anaerobic glycolysis. Along with mitochondrial renewal, this significantly contributes to myocardial energy recovery, thus slowing the progression of heart failure [56]. Eventually, the improvement of energy metabolism not only results in fuel supply but also facilitates the fitness of cardiomyocytes, reducing oxidative stress, cytotoxicity, and inflammation, thus attenuating adverse cardiac remodeling [1,56].

Recent studies have also suggested that GLP1-RA may have a potential visceral fat-targeting effect. In mice, their administration promoted brown adipose tissue thermogenesis and adipocyte browning, independent of nutrient intake [40]. These findings are particularly interesting since the GLP1 receptor is overexpressed in the visceral adipose depots of obese patients [40]. However, the mechanism underlying the actions of GLP1 and its analogs on adipose tissue remains unclear and requires further investigation.

In summary, the evidence suggests that SGLT2i and GLP1-RA induce compensatory metabolic changes starting with increased circulating ketone bodies that lead to beneficial mechanisms, including improved cardiac energy and efficiency. These findings strongly support the potential of these drugs, particularly SGLT2i, as protectors of the failing heart.

Overweight and obesity are serious medical conditions and a leading preventable cause of death worldwide, with rates increasing in both adults and children [65]. Emerging investigations are confirming the clinical utility of SGLT2i and GLP1-RA in the prevention and treatment of metabolic disorders beyond diabetes, including obesity [60]. As previously said, SGLT2i promote ketogenesis, fat browning, and a metabolic shift towards FFA utilization, which overall contribute to adipose tissue depletion and weight loss.

On the other hand, targeted trials have reported that GLP1-RA are also effective at reducing body weight and are being further investigated for their mechanism of action [66]. GLP1-RA exert a glucose-lowering effect, leading to a reduction in blood glucose and an improvement in postprandial glucose metabolism [67]. Additionally, the stimulation of GLP1 receptors in hypothalamic neurons provides satiety signaling, contributing to weight loss [67].

These findings provide additional evidence that SGLT2i and GLP1-RA are active metabolic regulators that create favorable conditions for cardiovascular protection beyond glucose control.

## 3. Peripheral-Vascular Protection

There is a growing body of evidence that suggests cardiometabolic drugs have beneficial effects not only on the heart but also on the vascular system, particularly in the context of peripheral artery disease [68].

SGLT2i have been shown to reduce cytokines, chemokines, and adhesion molecules associated with atherosclerosis [69]. Specifically, they inhibit pro-inflammatory cytokines, such as IL-1β, IL-6, and TNF-α, as well as attenuate NLRP3 inflammasome activation [70,71]. The expression of vascular-recruiting molecules, such as monocyte chemoattractant protein-1 (MCP-1) and vascular cell adhesion molecule-1 (VCAM-1), is also reduced [72]. Furthermore, SGLT2i promote plaque stabilization by reducing the activity of metalloproteinase-2 (MMP-2) and overall improving vascular fitness [73]. In diabetic murine models, SGLT2i prevent endothelial dysfunction by reducing arterial stiffness and endothelial impairment. Additionally, they balance neointima formation during atherogenesis by stabilizing smooth muscle cell abnormal proliferation, contributing to the preservation of correct contractile and microvascular function [74].

In addition, it has been demonstrated that the SGLT2i not only attenuate macrophage recruitment and infiltration but also favor M1 to M2 phenotype macrophage polarization, thereby reducing the inflammatory and oxidative environment and preventing foam cell formation [75].

Treatment with SGLT2i not only attenuates inflammation but also reduces indicators of oxidative stress in both the cardiomyocyte and vascular environment, leading to a restoration of nitric oxide (NO) bioavailability and providing vascular protective effects [69,76]. Controlled inflammation and oxidative stress presumably reduce platelet activation. Additionally, for patients with T2DM treated with SGLT2i, lower levels of plasminogen activator inhibitor-1 (PAI1) have been observed, which may help to deter thrombosis and stabilize plaque, further explaining the cardiovascular protection provided by SGLT2i [77].

On the other hand, GLP1-RA have been found to provide antiatherogenic effects beyond their glucose-lowering and weight-loss effects. They improve lipid parameters by lowering total cholesterol, LDL cholesterol, and triglyceride concentrations and increasing HDL cholesterol levels [78]. Additionally, GLP1-RA decrease high-sensitivity C-reactive protein levels and improve endothelial dysfunction, leading to better vascular fitness [78].

Another interesting effect of SGLT2i and GLP1-RA, contributing to cardioprotection, is related to the reduction of blood pressure [79]. Insulin resistance is considered one of the main causes of endothelial dysfunction, leading to reduced endothelium-dependent vasodilatory responses and, subsequently, hypertension [80]. Hyperinsulinemia is another factor contributing to increased blood pressure that causes fluid retention by increasing sodium reabsorption in the distal tubules of the kidney, which increases circulating plasma volume. In addition, hyperinsulinemia causes vascular remodeling by promoting the proliferation of vascular smooth muscle and other cells, thereby reducing vascular stiffness [81]. Few clinical trials have used blood pressure as the primary endpoint, and the results showed that both SGLT2i and GLP-1RA reduced blood pressure, indicating that the antihypertensive effect of SGLT2i may be slightly higher than that of GLP1-RA [82,83]. Indeed, the combination of antihypertensive drugs and SGLT2i in patients with diabetes improved blood pressure control more than the combination with GLP1RA [82,83]. SGLT2i lowered blood pressure steadily over a 24 h period, thus ameliorating nocturnal hypertension, which is mainly associated with cardiovascular risk, and nowadays, this peculiar effect attracts particular interest [84]. While the mechanism by which SGLT2i reduce blood pressure is still unclear, it is presumed that SGLT2i act mainly by reducing circulating plasma volume through osmotic and natriuretic diuresis in the early stages of administration and subsequently suppressing sympathetic nerve activity in the long term [85]. Differently, GLP-1RA show a lack of diastolic blood pressure-lowering effects and a slight increase in heart rate in the early stages of administration [50,85]. Instead, it has been demonstrated that GLP-1RA intake acts on weight loss through appetite suppression [86] and antiproliferative actions in vascular smooth muscle cells [87], which in turn could contribute to blood pressure reduction. Other factors that may contribute to blood pressure reduction are the anti-inflammatory effect and the role of Na^+^/H^+^ exchange on the proximal tubule [88].

Furthermore, emerging evidence suggests that SGLT2i and GLP1-RA provide significant therapeutic benefits in patients with pulmonary hypertension by favoring recovered hemodynamics in the right ventricle and pulmonary vascular remodeling [89].

Therefore, both SGLT2i and GLP1-RA have broad therapeutic prospects in the vascular context, provided their pharmacological mechanisms are further elucidated in future studies.

## 4. Future Perspectives

### 4.1. Dual-Receptor Agonist

This review highlights the close relationship between T2DM and multiple cardiometabolic diseases, such as heart disease, overweight, obesity, hyperlipidemia, hypertension, and various vascular complications. SGLT2i and GLP1-RA have demonstrated their efficacy in the treatment of these multiple-front cardiometabolic impairments, with significant therapeutic potential that can be further explored.

**Figure 2:**
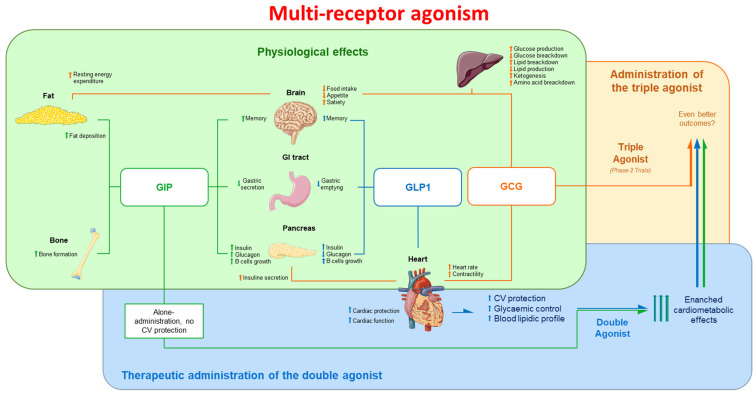
Multi-receptor agonists. The green box and arrows represent the action of GIP, the blue box and arrows represent the action of GLP1, while the orange box and arrows represent the action of GCG. The arrow pointing downwards reflect a downregulation of the mechanisms, while upwards arrows indicate an upregulation of the mechanisms. CV: cardiovascular; GIP: glucose-dependent insulinotropic polypeptide; GCG: glucagon; GLP1: glucagon-like peptide 1.

Despite this progress, ongoing research is evaluating new pharmaceutical options to aim for even more promising outcomes. One of these innovative therapeutic strategies is represented by dual-receptor agonists. These agents have the ability to activate both the GLP1 and glucose-dependent insulinotropic polypeptide (GIP) receptors, leading to enhanced glucose control, weight loss, and improvements in cardiovascular risk factors such as blood pressure and lipid profiles [90]. Several dual-receptor agonists are currently in development, and early clinical studies have shown promising results [91] (Figure 2).

Indeed, GLP1 and GIP receptor agonists belong to the same receptor family and regulate insulin release. GLP1 acts directly on the endocrine pancreas, gastrointestinal tract, brain, and heart, while GIP acts on the endocrine pancreas, fat, bone, gastrointestinal tract, and brain [92]. Both compounds bind to their respective receptors on pancreatic β-cells, stimulating insulin secretion glucose-dependently [92,93]. The targeted effects of these compounds on specific tissues make them a promising choice for the treatment of T2DM, with potential benefits beyond glycemic control, such as weight reduction and cardiovascular outcomes.

Indeed, GIP receptor agonists were initially thought to have no potential as a glucose-lowering therapy due to a lack of significant evidence of insulinotropic effects in individuals with T2DM [94]. However, emerging evidence has revealed that co-administration of GLP1 and GIP has a synergistic effect, resulting in a significantly increased insulin response compared to separate administration of each hormone. This has led to the development of tirzepatide, a novel dual GIP/GLP1 receptor agonist [94].

Tirzepatide was first approved in the USA in the spring of 2022 to enhance glycaemic control in adults with T2DM, in combination with diet and exercise. Currently, it is in phase III development for treating heart failure, obesity, and cardiovascular disorders in individuals with T2DM [95]. Notably, apart from its primary focus on diabetes, this medication has demonstrated remarkable cardiometabolic outcomes in ongoing clinical studies. Pre-clinical, phase 1, and 2 trials have indicated that this dual-agonist exhibits superior glucose and body weight reduction compared to using a single selective GLP1-RA alone. The adverse effects associated with tirzepatide are similar to those seen with GLP1-RA [96], ranging from mild to moderate gastrointestinal events such as nausea, diarrhea, and vomiting, with no clinically significant hypoglycemia [97].

Furthermore, alongside the reduction in body weight, notable improvements were observed in other related aspects [98]. The weight loss achieved with tirzepatide in T2DM patients was accompanied by enhanced glycemic control, a healthier blood lipid profile, and reduced blood pressure [99]. The drug demonstrated a significant reduction in the number of subjects affected by metabolic syndrome, which encompasses a combination of high-weight-associated cardiovascular risk factors such as obesity, hypertension, high triglyceride levels, low HDL cholesterol, and impaired glucose tolerance [98].

Overall, patients with T2DM treated with this dual agonist experienced a reduced incidence of major adverse cardiovascular events, including cardiovascular death, myocardial infarction, stroke, and hospitalization for unstable angina [100]. These outcomes bring significant hope for the future. Dual-agonists present promising prerequisites to emerge as a primary choice for the treatment of diabetes, offering substantial cardiovascular benefits while exhibiting relatively low-risk side effects.

However, it is important to note that these findings are still in the early stages, and phase 3 clinical trials will play a crucial role in ensuring the long-term safety of these compounds and confirming their cardiometabolic advantages. These trials will provide essential insights into the effectiveness and potential risks associated with the use of tirzepatide and similar dual agonists in a larger population. It is imperative to continue rigorous research and monitoring to fully understand the benefits and risks associated with these medications before widespread adoption can be recommended.

In conclusion, tirzepatide has shown impressive results in improving glycaemic control and promoting weight loss in individuals with T2DM. Moreover, it has demonstrated positive effects on other metabolic factors and cardiovascular outcomes. While these findings are promising, further investigation is necessary to ensure the long-term safety and efficacy of dual agonists like tirzepatide. Nevertheless, these compounds hold great potential to become a leading choice in the treatment of diabetes, offering significant cardiovascular benefits alongside manageable side effects.

### 4.2. Triple-Receptor Agonist

The high morbidity and mortality rates associated with T2DM highlight the growing need for even more effective therapies. Multi-receptor agonists are emerging as promising options for improving short-term and long-term outcomes related to hyperglycemia and obesity. In this regard, a novel compound called retatrutide has been developed. This single peptide acts as a triple-hormone-receptor agonist, capable of binding to GIP and GLP1 receptors (similar to tirzepatide) as well as the glucagon (GCG) receptor [101] (Figure 2).

Early studies have already assessed the safety, pharmacokinetics, and pharmacodynamics of retatrutide [102]. The drug demonstrated an acceptable safety profile, with mild to moderate gastrointestinal disorders being the most frequent adverse events, similar to dual receptor agonists [101,102]. Retatrutide administration showed robust glucose and body weight reductions, paving the way for phase 2 evaluations [102]. Subsequently, retatrutide entered a phase 2 clinical trial, aiming to develop it for the treatment of T2DM, obesity, and related comorbidities [101,103]. The outcomes of this trial revealed a significant weight reduction in the subjects receiving retatrutide, accompanied by notable improvements in cardiometabolic parameters, including systolic and diastolic blood pressure, glycated hemoglobin, insulin levels, fasting glucose, and lipid profile. The trial enrolled 338 adults, and the long-term testing period (48 weeks) substantially reduced body weight among obese patients [101]. Furthermore, a high percentage of participants with prediabetes experienced a reversion to normoglycemia with retatrutide treatment.

It has been speculated that the activation of the GCG receptor might further enhance the cardiometabolic effects induced by GLP1 or GIP–GLP1 agonism, particularly in terms of managing energy resources and related homeostatic functions [101]. In addition, a phase 2b, double-blind study was recently announced to examine the effect of retatrutide on renal function in participants with overweight or obesity and chronic kidney disease with or without type 2 diabetes (NCT05936151). This study will help us understand if retatrutide may have a protective property toward renal function; however, the mechanisms of action for this particular effect are still unclear.

However, the promising effects of retatrutide, such as clinically significant improvements in glycemic control and robust reductions in body weight likely leading to improved cardiometabolic outcomes, need further confirmation through phase 3 clinical trials to fully investigate the efficacy and safety of this new drug for the treatment of diabetes and obesity.

## 5. Conclusions

In conclusion, our study on SGLT2i and GLP1-RA highlighted their promising potential for improving cardiovascular health. Delving into the latest research, we have unveiled a multifaceted impact on the heart, physiology, and metabolism, extending beyond their conventional glucose-lowering role in patients with T2DM.

The impressive cardiometabolic outcomes observed encompass a spectrum of beneficial effects, ranging from improvements in energy homeostasis and substantial weight loss to notable reductions in blood pressure. Moreover, experimental and clinical data unveiled the substantial impact of these drug classes on mitigating oxidative stress, inflammation, cardiac fibrosis, and remodeling—crucial factors in the progression of cardiovascular diseases.

The collective evidence presented in this review underscores the potential of SGLT2i and GLP1-RA as integral components in the multifaceted management of cardiovascular health. As we continue to unravel the intricate mechanisms behind these effects, these drugs hold promise not only for individuals with T2DM but also for a broader population at risk of cardiovascular complications. Future research endeavors should delve deeper into the long-term effects and potential synergistic actions of SGLT2i and GLP1-RA, paving the way for innovative therapeutic strategies in the realm of cardiovascular care.

## Figures and Tables

**Table 1:** Summary of SGLT2i and GLP1-RA effects in experimental models and clinical trials.

	SGLT2 Inhibitors (SGLT2i)	GLP1 Receptor Agonists (GLP1-RA)
	Oxidative stress	
Experimental model	- Decrease apoptosis - Improve NOS activity - Increase NO production - Cytoprotective properties in endothelial cells - Stimulate Nrf2/ARE signaling - Suppress the TGF-β/Smad pathway - Prevent hydrogen peroxide formation - Preserve ATP production - Reduced the expression of NADPH	- Reduce neutral lipid accumulation, intracellular and mitochondrial ROS generation - Ameliorate IL-1β-induced ROS production - Suppress NOX-4 with a concomitant increase in superoxide dismutase 1 (SOD-1) and glutathione peroxidase - Reduce myocardial triglyceride and diacylglycerol levels through activation of the AMPK-Sirt1 pathway
Clinical Trail	- Decrease 8-iso-PGF2α - Reduce (NOX2)-derived peptide	- Decrease 8-iso-PGF2α plasma level
	Cardiac inflammation and fibrosis	
Experimental model	- Reduce extracellular matrix remodeling response - Decrease the expression of profibrotic markers - Lower cardiac and peripheral activation of NLRP3 inflammasome	- Reduce cardiac hypertrophy and myocardial fibrosis, lowering atrial weight, natriuretic peptide levels, and lung congestion - Reduce collagen deposition - Protect the heart against ischemic left ventricular dysfunction and improve the recovery of function during reperfusion - Arrest adverse cardiac remodeling after myocardial infarction
Clinical Trail	- Reduce left ventricular mass and beneficial outcome on diastolic function	- Not available
	Arrhythmia and fibrillation	
Experimental model	- Increase diuresis, glucose, and sodium excretion, resulting in a reduction of plasma volume, atrial blood pressure, and atrial dilation effects - Suppress the sodium-hydrogen exchange, reduce cardiac intracellular sodium and calcium levels - Reduce myocardial hypertrophy, fibrosis, and adverse remodeling, thereby reducing the risk of arrhythmia	- Not available
Clinical Trail	- Strong protection against incident AF/AFL while not improving stroke outcomes	- Reduction in stroke but does not significantly prevent the onset of AF/AFL
	Metabolic changes	
Experimental model	- Induce a metabolic shift towards ketone bodies, associated with polarization of M1 to M2 macrophages in adipose tissue, along with a significant fat-browning effect - Improve ketogenesis that may provide extra fuel, increasing cardiac efficiency - Reduce glucose transport within cells and contribute to intracellular nutrient-deprivation signaling - Promote autophagy as a systemic cellular defense to the starvation condition - Induce a recovery shift of metabolism from glucose to FFA, enhancing their transport and β-oxidation in the heart	- Promote brown adipose tissue thermogenesis and adipocyte browning independent of nutrient intake - Stimulate the GLP1 receptors in hypothalamic neurons, providing satiety signaling and contributing to weight loss
Clinical Trail	- Promote ketogenesis, fat browning, and a metabolic shift towards FFA utilization, which overall contribute to adipose tissue depletion and weight loss	- Reduce body weight - Improve the postprandial glucose metabolism
	Peripheral-vascular protection	
Experimental model	- Reduce cytokines, chemokines, and adhesion molecules - Inhibit pro-inflammatory cytokines, such as IL-1β, IL-6, and TNF-α, as well as attenuate NLRP3 inflammasome activation - Promote plaque stabilization by reducing the activity of MMP-2 and overall improving vascular fitness - Prevent endothelial dysfunction by reducing arterial stiffness and endothelial impairment - Balance neointima formation during atherogenesis by stabilizing smooth muscle cell abnormal proliferation	- Provide antiatherogenic effects beyond its glucose-lowering and weight-loss effects - Antiproliferative actions in vascular smooth muscle cells - Anti-inflammatory effect and the role of Na^+^/H^+^ exchange on the proximal tubule
Clinical Trail	- Lower blood pressure stably over a 24 h period, including night periods	- Lack of diastolic blood pressure-lowering effect and a slight increase in heart rate in the early stages

## Data Availability

The manuscript entirely reports published data. Spreadsheets with data as compiled for the present manuscript can be made available on reasonable request to the corresponding author.
